# A Study of the Experience of Working Adults on Regular Choral Activities: Applying Focus Groups

**DOI:** 10.3390/ijerph17238860

**Published:** 2020-11-28

**Authors:** In Hwa Shim, In Ok Sim

**Affiliations:** 1 Department of Culture Planning, Sung Kong Hoe University, 08359 Korea; alswl57@hanmail.net; 2 Red Cross College of Nursing, Chung-Ang University, Seoul 06974, Korea

**Keywords:** choral activity, experience, focus group, working adults

## Abstract

Maintaining a high quality of work and life can be a challenge for working adults. Most working adults do not have the opportunity to participate in activities that promote physical and emotional health. Working adults need to improve their potential to maintain life satisfaction and prevent a variety of stresses and physical illnesses. Recent research has suggested the effective value of art participation through choral activities for many people. However, little is known about what working adults experience through choir activities. In particular, research focusing on effects of choir participation on healthy people is lacking, and there is insufficient fundamental evidence about how to develop choral programs to improve the quality of healthy people’s lives. The purpose of this study was to explore the experience of working adults in the process of regularly participating in choral activities. Our research question is “What experiences did the participants gain from the choir activities?”. Data was collected in three focus group interviews and additional individual interviews with 15 participants. Four themes emerged from this study: “self-improvement and sense of accomplishment”, “interaction and harmony”, “discovery of positive self-identity”, and “healing and happiness”. These findings could encourage choir activity in working adults and suggest that they can be applied as lifelong activities that can improve their quality of life with social interaction.

## 1. Introduction

Maintaining a high quality of work and life can be a challenge for working adults. In particular, most working adults do not have the opportunity to participate in activities that support them physically and emotionally to stay healthy. In order to maintain an effective work life, working adults need to maintain personal and good lifestyle habits. It is especially important to have the opportunity to develop self-esteem, expand overcoming thinking, and develop capacity to improve your potential. These results can reduce conflict and stress from work in the workplace, improve work efficiency, prevent physical illness, and experience self-satisfaction and happiness [1]. Increasingly, many people recognize that their personal core values include enjoying social activities to achieve happiness [2]. Their sense of happiness derives from evaluating their satisfaction with their own existence rather than comparing themselves to others [3]. Participating in art and cultural activities can help maintain high quality of life, positive thinking, and happiness. It also promotes self-awareness, self-realization, and self-improvement, which allows people to express themselves freely and provides opportunities for social integration [4]. Additionally, such experiences allow individuals to understand and appreciate the innate beauty of human nature. Positive and creative thinking arises from joyful, beautiful, and good-natured actions expressed through culture and art [5]. The artistic world offsets the competition inherent in the social structure, with its dominant–subordinate relationships; by respecting each individual’s individuality and uniqueness, the arts encourage a subjective and communal life.

The experience of music activities contributes to improving the quality of life by increasing life satisfaction, self-efficacy, personal happiness, and reducing stress and anxiety levels [2,3,6]. Sim [7] reported that through regular choral activities, nursing students discovered identities and strengthened positive interpersonal relationships, especially by strengthening social adaptation and confidence. Furthermore, choral activities strengthen communication skills and allow people to freely express their feelings, allowing them to participate in society [8]. Previous studies to date have demonstrated the effectiveness of choral activities for elementary school children, adolescents, middle-aged women, and the elderly. In addition, studies using therapeutic choral activities were conducted on adolescents who experienced violence against children and patients with chronic diseases such as depression, autism, and dementia [2,4,6,7].

Most of the research results explain the positive effects of music, but it is still applied only to limited research subjects, and specific studies focusing on the effects of music activities for health promotion and disease prevention of healthy adults are insufficient. Moreover, there is insufficient rationale for the in-depth experience of working adults after participating in regular choral activities. 

The purpose of this study was to explore the experiences of working adults who are regularly participating in choral activities, and what they perceive in the course of their lives. 

Therefore, through this study, we intend to present basic data so that various choral activity programs can be developed that can be helpful to working adults in the future.

### Research Questions

The study aimed to examine the experience of regular choir participation on working adults. Our research question was: What experiences did the participants gain from the choir activities? 

## 2. Materials and Methods 

### 2.1. Design

This study was a qualitative study that applied the analysis method of the research model developed by Colaizzi [9] as a phenomenological research method to understand and explore the experiences of working adults through choral activities. Focus groups consisted of a small number of participants, typically with similar characteristics. An advantage of this methodology was easier understanding of the targeted phenomenon; particularly, it allows for better understanding of the motivations behind people’s behavior and experiences [10,11]. We used this method to conduct an unstructured discussion with a group of people with a common interest and promote group interactions by describing their experiences and understanding of the situation. 

### 2.2. Participants and Settings

Participants were selected as adults working in Seoul and Gyeonggi Province, who joined the choir and had experience in regular choral activities. In order to recruit participants, the purpose of the study was announced to those who participated in the choral activity as working adults through email, social network service (SNS), and acquaintances, and the subjects who would like to participate in this study were selected. There were 18 participants who finally agreed to participate in the study, and in this study, each group consisted of 6 subjects and 3 groups. According to Morgan and Scannell [12], this is the most effective for focus groups, with 6–10 people per team, suggesting that it would be appropriate to organize them into 3 groups so that they could sufficiently present a variety of opinions in the discussion. However, in the interview process, the final 15 people participated, excluding 3 participants who were unable to continuously participate in the focus group interview. The 15 participants who were doing choral activity at least once a week in a choir; interviews were conducted after participants were divided into three focus groups of four, five, and six people. 

### 2.3. Ethical Considerations

All participants were approached individually by the researchers and participated voluntarily after having thoroughly understood the study’s purpose and methodology. The study was conducted after receiving approval from the Chang Ang University Institutional Review Board (1041078-201903-HRSB-090-01). Informed consent was obtained from all participants, who were aware that they could withdraw without giving a reason at any time without repercussions.

### 2.4. Data Collection

This study was conducted from 10 June to 30 September 2019 for data collection. Participants received detailed information on the purpose, content, and rights of the participants in this study, then filled out a consent form, and explained the recording of interview content, confidentiality of personal information, and discontinuation of participation in the study. The researchers conducted a semi-structured interview guided by a set of prepared open-ended questions. Focus groups consisted of four to six participants, which meant that each participant had plenty of time to speak, creating an open atmosphere in the discussion sessions.

All sessions were guided by open-ended questions, specifically about how participants had come to join the choir, any difficulties they had experienced and possible ways to solve them, effects of choir participation, specific changes that had occurred in their lives, and their plans for personal and social development through the activity. Focus group interviews ranged from 100 to 180 min but lasted 100 min on average. Additional data were collected using individual interviews and closed-ended survey questions when there was a need for deeper understanding of a particular personal experience with choir participation. All interview sessions were conducted either in seminar rooms at an institute affiliated with the researchers or at any comfortable location that was convenient for participants. All interviews were recorded with interviewees’ consent.

### 2.5. Data Analysis

In the analysis process of this study, the analysis method proposed by Colaizzi [12] was applied to the data collected through focus interviews. As the first step in the analysis process, after repeatedly listening to interviews with researchers and describing the participants’ statements in writing, meaningful statements were extracted. Next, by grasping the clear meaning of the extracted meaningful statements and describing them in the language of the researcher, the subject and the subject cluster were formed and integrated to comprehensively described the intrinsic structure. 

In order to increase reliability and validity, the final analyzed results were presented to the study participants to ensure that the results well explained the meaning of the original data and matched the participants’ experiences.

To follow the four evaluation criteria proposed by Lincoln and Guba [13]: True value, applicability, consistency and neutrality, how accurately the participant’s experience was actually described, and whether the researcher’s explanation was consistent with the participant’s experience was repeatedly checked. In addition, two authors of this study with experience in qualitative research conducted an in-depth analysis of the data collected until no new data were available. In order to increase the reliability of the study, the interview contents and statements were checked, and to secure validity, the final analysis was conducted by cross-evaluating with two professors who are familiar with qualitative research.

## 3. Results

### 3.1. General Characteristics

The average age was 45 years (range: 30 to 59). There were seven men (47%) and eight women (53%); eight participants were married (40%) and seven unmarried (47%). Nine participants (60%) had between six months and a year’s experience of participation in the choir, while six (40%) had more than a year’s experience. Six participants (40%) participated in a group rehearsal more than once per week, while nine participants (60%) did so more than twice per week. In terms of motivations for participation, 13 people (87%) reported participating through their own initiative, while two participants (13%) reported that they participated because they were encouraged or recommended to by others. These characteristics are listed in Table 1. 

### 3.2. Results Based on Colaizzi Analysis

Analysis of the significant statements regarding the experiences through regular music were grouped into the following four themes: (1) “self-improvement and sense of accomplishment”, (2) “interaction and harmony”, (3) “discovery of positive self-identity”, and (4) “healing and happiness” in Table 2.

#### 3.2.1. Theme 1. Self-Improvement and Sense of Accomplishment

The theme of self-development and sense of accomplishment included sub-themes such as improving own integrated abilities and processing the achievements. 

While participating in the choir, participants experienced difficulties, tiredness, and frustration, and repeatedly made mistakes during practice; despite all these challenges, they pushed themselves to keep the promise they had made to one another and to build trust with other choir members. Even if such efforts went unacknowledged, they represented the participants’ driving motivations and their desire to satisfy their expectations of themselves. 

As people age, they sometimes give up on their dreams, which can cause feelings of shame or lethargy. Choir participation, however, helped participants to overcome such feelings. In particular, they mentioned that after a concert, which represented the fruit of all their efforts, they felt a sense of accomplishment and discovery of their own potential. The participant’s statements describing the sub-themes corresponding to this theme are as follows. 

##### Sub-Themes 1. Improving One’s Own Integrated Abilities

Depending on how you interact with people through music-related activities, you can improve yourself in whatever way you want, and even though choir practice is difficult, it is a pleasant stress to overcome.

(Participant 1)

I have learned to recognize a way of life that is more predictable than before through stable relationships, practice, and the concert. … It is as if I have seen potential in myself? … I am thinking that facing a challenge could be stressful but also might be a great opportunity for a positive outcome…

(Participant 3)

Even though I know that we are amateurs, we have this common goal of making this concert close to perfect, and in the process of achieving the goal, we improve our musical skills and our ability to manage conflict is strengthened.

(Participant 12)

##### Sub-Themes 2. Processing the Accomplishment

It is not easy for someone without a specialization to create and present an artistic performance, but we were able to do it even though we were not specialists. It means that it takes a lot of time and effort to acquire a certain level of skill to produce harmony, but the sense of fulfilment was a great thrill.

(Participant 15)

There is a sense of accomplishment that comes from the workplace, but I think it is much bigger when it comes from a hobby in the arts and culture, when I do it voluntarily. Experiences in the arts and cultural activities make life more relaxed, so they allow one to have lively energy, a life with a little bit of tension and new ideas.

(Participant 10)

#### 3.2.2. Theme 2. Interaction and Harmony

The theme of interaction and harmony included sub-themes such as realization of interaction and creating harmony. Choir participation helped individuals share their ideas and create relationships with each other through harmony. In this process, the choir developed musical and communal relationships and acquired a deeper understanding of the group as a whole. Choir allowed participants to share each other’s interests, and through this process, they could build a sense of cooperation and a strong bond through minds and voices, rather than with language, creating true harmony with each other. The participant’s statements describing the sub-themes corresponding to this theme are as follows. 

##### Sub-Themes 1. Realization of Interaction

I think the basis of choir practice lies in putting an effort into forming human relationships in which you recognize and assist each other, and I think this can guarantee a sense of engagement and accessibility. This is basically about making sounds you want together.

(Participant 10)

I tend to look back on myself with a broad perspective and have time to restrain my feelings when I am having trouble with choir practice, and when someone looks down on me; and in this way, I learn to put up with my feelings to maintain relationships with others. I also learn that there are many things that I do not know when I practice. I realized that to learn things that I do not know, I should socialize with good people, too!

(Participant 6)

The process of tuning in to each other in music is democratic, and there is some respect in it. In addition to the direct experience of the chorus, this trust in lasting human relationships lowered the guards between people and made me feel satisfied.

(Participant 9)

I’m a member of the choir, and I have the opportunity to explore new career paths through relationships with the people around me. Also, I feel like I have a lot of opportunities to have deep human relationships.

(Participant 5)

##### Sub-Themes 2. Creating Harmony

Through our songs, we naturally understand the center of our hearts and love each other more without feeling the differences in what we do individually. In order to create a good harmony, we have to keep each other in mind and wait until we understand each other through the chorus. It is also possible to develop the ability to listen to each other’s stories and communicate one’s own story.

(Participant 15)

The exquisite harmony of music gives us a special feeling of happiness, and we experience increased mutual engagement. I also learned that I cannot do anything with only my own thoughts and actions; the harmony cannot be created with just my own actions and I cannot live in my own way.

(Participant 2)

#### 3.2.3. Theme 3. Discovery of Positive Self-Identity

The theme of discovery of positive self-identity included sub-themes such as real self-awareness through music and changes in positive thinking about others and self. Another aspect of choir participation was the discovery of a positive self-identity through self-reflection. Participation in a choir is a process of making beautiful music as a collective, in doing so, participants reported discovering the “good-natured aspect” of themselves and recognizing their own “sincerity” and “true self.” They had the opportunity to self-reflect and discover what was wrong and lacking, especially when obstacles needed to be overcome during practice. Common statements relating to changes in thoughts about oneself in the process of creating music referred to the perception that the “existing framework is broken” and that “I am different with others.” Furthermore, participants reflected on themselves by saying “I do my best to create beautiful harmony.”. The participant’s statements describing the sub-themes corresponding to this theme are as follows. 

##### Sub-Themes 1. Real Self-Awareness through Music

When I was practicing singing, I felt sincere to myself, and the sound of music made me feel other people’s heart and sincerity … Choir practice felt like participating in holy places like churches and spiritual repentance, such as prayer. I became more considerate and prosperous in life than when I used to guard against people.

(Participant 2)

The chorus comes together with good minds and will. Through sounds, I felt that everyone taking part in the music was good-natured and I recognized my true self and sincerity. Since I think that there is no malice in negative situations or opinions, I learned to manage problems: problems can become different when you change your perspective.

(Participant 6)

It is not that all artistic activities as part of the choir are right, fun, and everyone should do them, but one thing for sure is that there are bad and good times in it. And it is a valuable experience in life to see myself being different from others.

(Participant 9)

##### Sub-Themes 2. Changes in Positive Thinking about Others and Self

We were able to take part in the activity with our true selves, and it made us see the innocent and sincere aspects of ourselves and others. I also felt that people in the choir were really good people, so I felt that I wanted to be with them. I believe that it was an opportunity to make myself a good person and think about myself as such.

(Participant 13)

Change through music specifically creates a feeling of being a mature human, and we remain sincere and innocent when participating in music.

(Participant 12)

When I tried my best to achieve a beautiful harmony, I felt that it was meaningful because it made me look at myself and others as pure and innocent as a child.

(Participant 14)

#### 3.2.4. Theme 4. Healing and Happiness 

The theme of healing and happiness included sub-themes such as being healing and feeling happy. During choir practice, most participants felt that their minds were being healed and experienced happiness through their musical activities. Despite suffering from stress, physical fatigue, and anxiety about their personal lives, participants said that musical activity helped them to recover from troubles and settle their minds, fostering inner peace and a happy life. During practice, participants regularly interacted with other members and adjusted their own voice to others’ voices to create a harmonious sound. As such, becoming a choir member formed a framework of human relationships in which an integrated sense of working with others was understood and accepted. This created a beautiful harmony of sound and maturity in human relationships. These results not only provided individuals with satisfaction and stability in their relationships, but also gave them the energy to find pleasure, excitement, and happiness. 

##### Sub-Themes 1. Being Healing

I thought that I liked being alone, but through this regular activity, I realized that things like meeting people, singing, going up on stage, and clapping for each other bring energy to my life.

(Participant 2)

I had always been overwhelmed and unstable in my life, but such feelings seemed to disappear when I come here. It seems to have the same effect of healing in nature by taking time off from the weight of everyday life.

(Participant 3)

##### Sub-Themes 2. Feeling Happy

I enjoy playing alone, but when I sing with others in chorus and we perform together at the front of the stage, I feel a sense of catharsis through the mystique of music in harmony.

(Participant 8)

The exquisite harmony, approaching perfection, seems to bring happiness, transitioning the state of depression into joy. I was able to find mental and physical health by looking back at myself in a holistic way, and I gained emotional stability through healing of my mind.

(Participant 13)

## 4. Discussion

### 4.1. Self-Improvement and Sense of Accomplishment

The most important part of participating in a choir is the process of creating harmony with others through music. The process of achieving harmony through music with others is not an easy process. Choral activity requires repetitive practice to tune different individual pitches that blend with other distinct pitches. Choral activities that require different individuals to follow the opinions of others and collaborate in order to achieve a perfect harmony of music can sometimes lead to fatigue and pressure. Nevertheless, it can be seen that the moment they overcome all difficulties and achieve a beautiful harmony through chorus activities, they experience a sense of confidence and achievement. In this way, the results obtained from the process of doing their best to maintain the trust and cooperation of the choir members were found to feel satisfaction, the inner conflict disappears, and a sense of accomplishment. These findings are also reported in Lee’s study [7,14] on achieving integrity through interventions and music therapy and challenges.

Considering these results, adults’ choral activity gives them the opportunity to recognize their own achievements and grasp their potential as individuals who can overcome problems, which can result in a leap forward towards self-development. This discovery also supports Sim’s findings [7] that performing arts lead to self-realization, helping individuals improve their interests and aptitudes. 

A study in which young people could participate in group musical activities showed that these activities increased self-esteem by providing opportunities for satisfaction and increasing individuals’ inner sense of worth [14]. Sim’s study [7] also confirmed that regular choir participation strengthened self-confidence and sense of achievement in college students. Therefore, it may be beneficial to provide the general public with opportunities to participate in musical activities, allowing them to develop themselves through a positive sense of fulfilment.

### 4.2. Interaction and Harmony

Choir participation helps others share their thoughts and build relationships, which involves developing a deeper understanding of other people. This process builds mutual cooperation and strong bonds through the sharing of hearts and harmonizing of voices. Through their relationships, people can self-reflect with a broad perspective. Being inspired by music creates a special sense of happiness in one another and increases mutual trust. As such, the importance of choir is to recognize that one cannot do anything alone. Harmony is dependent upon others. Therefore, choir harmonizes people’s relationships. 

Moreover, participating in a choir requires a deeper understanding of others, relationships that help others share and build their thoughts. In choral activities, people from different fields naturally unite, the problems are different, but they get rid of personal feelings, get to know each other, reach the goal of unity, and build a mature relationship and harmony with others. Conway and Hodgman [2] reported that choir participation created important human connections because choirs naturally integrate people from various fields, allowing them to learn about one another and maintain relationships while reaching a common goal. Additionally, as part of their role as a choir member, each individual has to learn their own musical part. In this process, members learn to focus on the practice by putting aside personal feelings and prejudices. Sometimes unexpectedly, stressful situations during practice lead to complaints or anger; however, with the goal of producing a single harmonious voice, individuals learn to deal with these situations and feelings, thus maintaining a good atmosphere and building mature relationships with other members.

Various studies show positive effects of musical activity in relation to harmony, interaction, and relationships [6,7,15]. Yang [6] reported that choir participation helped middle school students by strengthening their communication with friends. This finding is in line with the results of the present study. In one study [16], choir participation allowed participants to feel satisfaction when they were immersed in the harmony of sound and emotion. Sim [7] also showed positive effects on the process of collaboration through mutual relationships as a result of choir participation, thus supporting the research on a theoretical basis.

Singing during choir practice entertains the mind and allows individuals to channel energy through lyrics and rhythm. The greatest advantage of choir is that individuals feel the thrill of creating a harmonious voice through integration of multiple voices. Although invisible, this force can have positive effects on people’s minds. Choir participation allows individuals to interact with each other through music and feel a sense of unity. 

### 4.3. Discovery of Positive Self-Identity

Choir is an opportunity to discover a positive inner identity through creating full harmony. Practice encourages patience, coping skills, and positive thinking. In addition, practice allows one to recognize one’s shortcomings and repeat efforts to resolve them. As such, music is a tool for building a mature character. The positive effects of choir participation, such as self-reflection and discovery of one’s good nature, are supported by theory. Lee et al. [16,17] reported that people who participated in musical activities found their own identities and learned to engage in positive mental control, self-confidence, self-control, and self-management, thus discovering a passion for life; these results accord with the present findings. This study also found that reinforcement of self-reflection through music participation was fundamental in improving the challenging nature of one’s occupation and helping individuals to think ethically and morally.

### 4.4. Healing and Happiness

Common to all participants’ experiences was an increased quality of life and mental healing through music. Adults experience responsibilities, stress, and physical fatigue, but musical activities can help people maintain a happy life and active lifestyle [18,19]. Regular social interaction, the role of being a choir member, and beautiful harmony brings happiness, healing, and pleasure. This creates a beautiful blend of self-satisfaction and maturity in human relationships. As such, the results of this study reflect the findings of a study according to which chorus activities provide an opportunity to find freedom and happiness by reducing stress or anxiety and promoting mental healing and emotional stability [20,21]. 

These results are similar to Gul’s finding [3] that participation in choral activities makes physically and emotionally difficult situations more endurable and promotes happiness, peace, and enthusiasm. Furthermore, the results of the present study are also consistent with the decline observed in inherent depression when clinically depressed individuals participate in choral activities [22]. Participation in a choir enhances the quality of life experienced by the general public, which is similar to various effects of choir participation reported in preceding research. According to these results, choral activities are considered to have a positive effect and unique benefits for working adults than any other group activities.

The limitation of this study is to explain the subjective and experience effects of the study participants, so it is not sufficient to explain the causal relationship between music activities and various factors of office workers. Therefore, it is suggested that a quantitative study is needed to confirm the effect of various music activities on job satisfaction, quality of life, stress, or problem solving for working adults in the future.

## 5. Conclusions 

The results of this study explain the meaning of various positive aspects such as self-development and a sense of achievement, interaction and harmony, discovery of positive self-identity, healing, and happiness in the analysis of working adults’ experiences through chorus. These results provide a basic theoretical basis for what the empirical meaning is through participation in choral activities, the purpose of this study. In addition, it is possible to solve various problems that exist in working adults, stress and health problems caused by work, and to build positive egos and relationships, and for their own development, choral activities can be explained as very positive. 

The suggestion through the results of this study is to present the practice of quantitative research that can verify and confirm the theoretical basis for understanding and explaining experiences through choral activities in the future. In addition, since choral activities for vocational adults have consequences that can contribute not only to individuals but also to society, policy implementation is required to support them. The significance of this study suggests that it is necessary to provide opportunities to participate in music activities not only for working adults, but also for people with social problems, low-income groups, marginalized groups, and people with poor quality of life. In addition, it aims to emphasize the need to provide a social environment that supports choral singing as a policy measure to improve cultural welfare as well as include office workers.

## Figures and Tables

**Table 1:** Participant characteristics.

No.	Gender	Age (Years)	Occupation	Experience of Music Activity	Number of Participations in Practice/Month
1	Male	30	Culture center	1 year	5 times
2	Male	43	Social worker	1 year	5 times
3	Male	45	Culture planner	4 years	5 times
4	Female	58	Social worker	1 year	5 times
5	Female	38	Announcer	1 year	4 times
6	Female	49	Film production	5 years	5 times
7	Female	49	Painter	5 years	5 times
8	Female	50	Painter	5 years	5 times
9	Female	49	Teacher	5 years	5 times
10	Male	56	Lecturer	5 years	5 times
11	Male	34	Media researcher	1 year	4 times
12	Female	40	Novelist	1 year	5 times
13	Female	46	Theatre officer	1 year	4 times
14	Female	46	Teacher	1 year	5 times
15	Male	42	Officer	1 year	5 times

**Table 2:** Classification of themes.

Themes	Sub-Themes	Formulated Meaning
Self-improvement and sense of accomplishment	Improving one’s own integrated abilities	Learning to communicate with people through music
		Overcoming difficulties in life through practice and concert
		Finding my potential ability
		Experiences in arts and cultural activities make life more comfortable, energetic energy, and discover new ideas
	Processing The accomplishment	Choral activity is more meaningful and a sense of accomplishment than at work
		Challenge is a stressful process, but it is a great opportunity to achieve positive results.
		Acquisition of skills for harmony in music takes a lot of time and effort, but it acquires a joyful and great sense of accomplishment.
Interaction and harmony	Realization of interaction	Chorus is an opportunity to build relationships that recognize and help each other
		The process of reconciling each other in music is possible when there is respect for the other person
		Learning to hold back my emotions in order to maintain relationships with others through music
		To create good harmony, we have to wait for each other to understand each other.
		As a member of the choir, you can have a deep human relationship through relationships with others.
	Creating harmony	The importance of chorus is basically to make the sound you want together
		They naturally understand and love each other through songs.
		The exquisite harmony of music gives us a special sense of euphoria and experiences that mutual participation increases
		Realizing that you can learn what you do not know when you hang out with good people
		My thoughts and actions alone are not in harmony with others, and I cannot live my way.
Discovery of positive self-identity	Real self-awareness through music	Recognize your authenticity when practicing singing
		Participating in choral practice creates a feeling of prayer and spiritual repentance
		Music is the process of making myself a good person
	Changes in positive thinking about others and self	The harmonious sound of music lets you know the hearts and minds of others
		Positive self-discovery of paying attention to others
		Through music, everyone is kind, and I find my true self and sincerity.
		Understanding the innocence of oneself and others through harmony of music
		Can turn negative situations into positive perspectives
Healing and happiness	Being healing	Unstable life and pressure disappear
		Free from the weight of daily life and has the effect of natural healing
	Feeling happy	Happiness in life to go on stage with people through music activities and clap for each other
		Feel happy through the mystery of harmonious music when singing chorus with others and performing together in front of the stage
